# Intermuscular Posterolateral Minimally Invasive Open Approach Revisited: Spectrum of Applications and Stabilization Outcomes

**DOI:** 10.7759/cureus.117048

**Published:** 2026-09-28

**Authors:** Ajay Krishnan, Bharatkumar R Dave, Amritesh Singh, Shiv Kumar Bali, Shivanand C Mayi, Ravi Ranjan Rai, Mirant B Dave, Mikeson Panthackel, Arjit Vashishtha, Yogenkumar Adodariya, Saurabh S Kulkarni, Preety Krishnan

**Affiliations:** 1 Spine Surgery, Bhavnagar Institute of Medical Science, Bhavnagar, IND; 2 Spine Surgery, Stavya Spine Hospital and Research Institute, Ahmedabad, IND; 3 Orthopedics, All India Institute of Medical Sciences, Rishikesh, IND; 4 Orthopedics, Mahatma Gandhi Medical College and Research Institute, Aurangabad, IND; 5 Radiology, Stavya Spine Hospital and Research Institute, Ahmedabad, IND

**Keywords:** miss, navigated pedicle screw, posterolateral approach, thoracolumbar instability, weaver's approach

## Abstract

Introduction and aim: Conventional posterior midline open surgical approach (COA) for thoracolumbar spine fixation requires extensive muscular dissection and retraction, resulting in significant complications, including excessive intraoperative bleeding, prolonged postoperative pain, muscle ischemia, and delayed functional recovery. While percutaneous pedicle screw fixation (PPSF) offers reduced invasiveness, it carries inherent risks of radiation exposure and high rates of pedicle screw breach. The posterolateral intermuscular minimally invasive open approach (PLOA) represents an alternative MISS technique that develops an intermuscular plane between paraspinal muscles. This study aimed to evaluate the safety, efficacy, and clinical utility of PLOA pedicle screw fixation (PSF) in various thoracolumbar spine pathologies requiring stabilization without extensive decompression

Materials and methods: A retrospective analysis was conducted on 47 patients who underwent PLOA PSF between January 2016 and January 2023 at a tertiary care spinal unit and peripheral unit, with a minimum 24-month follow-up. Inclusion criteria encompassed traumatic fractures, fragility fractures, and pathological fractures, including spondylodiscitis and metastatic disease. Standard preoperative classification systems were applied for traumatic fractures, pathological fractures, and infectious cases. Surgical technique involved 8-10 cm paramedian incisions utilizing region-specific intermuscular posterolateral planes. Intraoperative variables, including operative time, blood loss, and screw accuracy, were documented. Postoperative assessment included hospital length of stay, analgesic requirements compared to COA, complications, and radiographic loss of correction on immediate and final follow-up radiographs using segmental Cobb's angles. Statistical analysis was performed using SPSS version 20.0 (Chicago, IL: SPSS Inc.).

Results: A total of 47 patients (mean age 59.1±12.04 years; 53%, n=25 males) were enrolled. Primary indications included traumatic fractures (38.2%, n=18), infective spondylodiscitis (36.2%, n=17), pathological tumors (12.7%, n=6), and fragility fractures (8.5%, n=4). A total of 289 pedicle screws were placed (mean 6.14±1.43 per patient), with 100% accuracy achieved in both fluoroscopic (51%, n=24 patients) and O-arm navigated (49%, n=23 patients) insertions. Mean operative time was 61.73±14.9 min and 59.56±11.06 min, with minimal blood loss of 44.78±8.45 mL and 45.21±8.45 mL. Hospital stay averaged 2.46±0.55 days. Postoperatively, 61.7% (n=29) of patients required less analgesia than COA patients, while 29.7% (n=14) required equivalent analgesia and 8.5% (n=4) required more. Loss of correction ranged from 0-20° (mean: 3.46±4.92°) in 30 patients (63.8%), while 17 patients (36.2%) maintained correction. Complications were minimal, with 95.7% (n=45) experiencing no adverse events.

Conclusion: PLOA represents a viable minimally invasive intermuscular dissection alternative for open thoracolumbar spine stabilization across diverse pathologies, demonstrating superior technical efficiency, minimal blood loss, and rapid postoperative recovery. It is a lower-morbidity approach in resource-limited settings where PPSFs per se are costlier or technology is unavailable in remote settings.

## Introduction

Conventional posterior midline open surgical approach (COA) is the most common method used for treating various spinal ailments requiring fixation with or without fusion in the thoracic and lumbar region. COA requires extensive stripping and excessive retraction of muscle from the midline to the transverse process for accurate placement of pedicle screws (PS). This leads to excessive intraoperative bleeding, pain, additional analgesics, blood transfusions, and transfusion-related complications. Moreover, loss of innervation, posterior ligamentous complex (PLC) injury, ischemia, muscle atrophy, scarring, and fatty replacement can manifest as a cause of delayed postoperative recovery, residual intractable back pain/stiffness/dysfunction, and suboptimal outcomes resulting in delayed/no return to work [1]. For spinal ailments requiring fixation with or without fusion, pedicle screw fixation (PSF) is the method of choice [2]. This further adds to excessive posterior midline dissection, and PLC injury plays a pivotal role in the early occurrence of adjacent segment disease (ASD) and proximal junctional kyphosis (PJK) [3]. To circumvent these problems, the focus of surgical management has shifted toward paraspinal, posterolateral, lateral, and even ventral approaches in the last three decades, which are more towards anatomical planes with a focus more evolved toward minimally invasive spine surgery (MISS). The evolving concepts have led to earlier recovery, including various aspects of ambulatory surgical care and enhanced recovery after surgery (ERAS) philosophies [4]. Furthermore, percutaneous pedicle screw fixation (PPSF) has evolved to be a safe and effective method of fixation, and results are comparable and even superior to PSF by COA [5]. PPSF depends on tactile feedback and on fluoroscopic image guidance. It is associated with a high incidence of pedicle screw breach and high doses of intraoperative radiation exposure [5]. Futuristic, precise Integrated Operation Theater Spine Suite (IOTSS) and specialized gadgets, though costly, are safe and have a steep learning curve [6]. Posterolateral intermuscular minimally invasive open approach (PLOA) is an alternative MISS technique for insertion of pedicle screws and is popularly known by more common names, such as Weaver’s approach in the lumbar spine [7]. Although open and with a combined incision length greater than COA, it follows the principle of MISS, which involves using approaches that should be defined not just by a skin incision, but rather by preserving the anatomy. It develops an intermuscular plane. This exposes the lateral part of the pedicle, the isthmus, transverse process, and the facet, thus giving direct exposure of the pedicle screw entry [7].

However, this exposure is not sufficient for extensive decompression. Wiltse and transmuscular approaches are more common approaches where central and cross-over decompressions are possible [8]. Though PPSF is the recommended and widely used method, the utility of PLOA can be explored in low-cost settings without high-technology enablers. Primarily, the authors report a retrospective analysis of the outcomes of PLOA PSF in various pathologies of the thoracolumbar (TL) spine needing only stabilization for instability. Secondarily, this article attempts to revisit the indications when decompression of the spine is not necessitated in the thoracolumbar spine.

## Materials and methods

All the patients who underwent spinal fixation by the PLOA PSF method and fulfilled the inclusion and exclusion criteria between January 2016 and January 2022 at our tertiary care spinal unit and peripheral unit were included in the study. We also included cases with a minimum of 24 months of follow-up, or until any mishap (in malignancy) before 24 months. The selection criteria were as follows: inclusion criteria included traumatic fractures, fragility fractures, pathological fractures of different etiologies, infective spondylodiscitis, and metastasis/primary tumor, and patients who were poorer and neither could afford a percutaneous screws system nor wanted to pursue the subsidized health schemes. Exclusion criteria included patients with incomplete records, those previously operated on at the same or adjacent levels, and patients who presented with other coexisting spinal, joint, metabolic, congenital, or other diseases that could significantly influence the patient outcomes. The primary objective of the study was to assess the perioperative and radiographic outcomes of the approach.

The patient flow diagram is depicted in Figure 1. All patients were assessed thoroughly, and demographic details (age, sex, and BMI), primary diagnosis, preoperative neurology, American Spinal Injury Association (ASIA) grading, classification of disorder, and spinal region were recorded. Different classification systems were used for objective preoperative scoring. Spinal Instability Neoplastic Score (SINS) was used in pathological fractures secondary to primary or secondary tumors [9]. The score ranges from 0 to 18. A score of 0-6 indicates stability, 7-12 indicates impending instability, and 13-18 indicates instability. Pola et al.'s classification system for pyogenic spondylodiscitis was used for cases of infection [10]. Classification was based on primary criteria such as extent of bony destruction or presence of segmental instability, epidural abscess, and neurological impairment. Furthermore, subclassification was done using secondary criteria like involvement of paravertebral soft tissues and intramuscular abscesses. Thoracolumbar Injury Classification and Severity Score (TLICS) was used in traumatic and osteoporotic fractures for deciding the management [11]. The score is based on the following three parameters: fracture morphology, integrity of posterior ligamentous complex (PLC), and neurological status. A score of 0-3 indicates non-surgical treatment and >4 indicates surgery.

**Figure 1:**
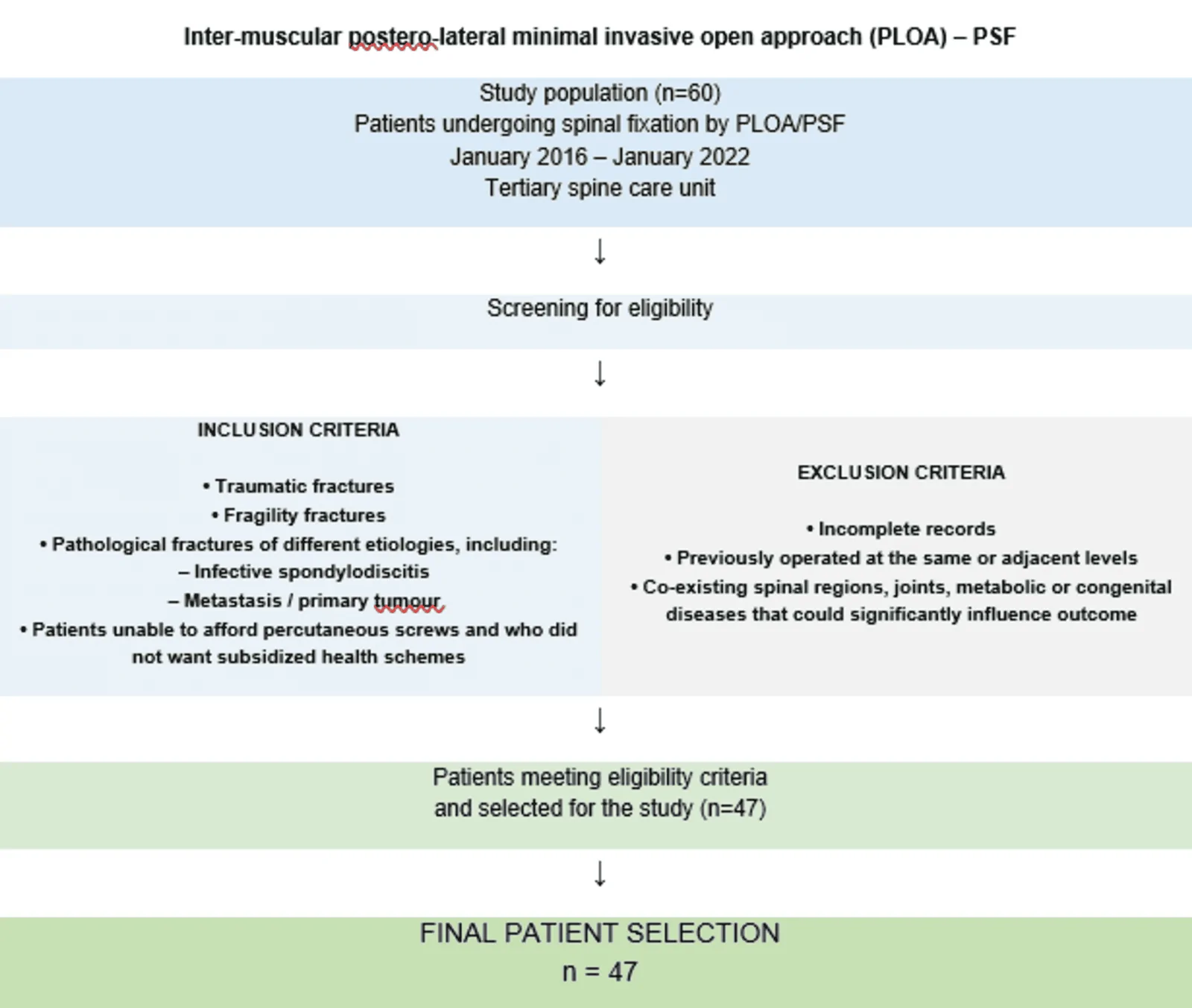
Patient selection flow diagram. PLOA: posterolateral intermuscular minimally invasive open approach; PSF: pedicle screw fixation

Operative technique

After general anesthesia, patients were placed prone on bolsters. Approximately 8-10 cm long paramedian incisions were made on either side of midline, 5-7 cm from midline, depending upon body habitus and region of surgery, for three segments and increased if more screws were needed. More medialization of the incision was done in the thoracolumbar region and in thinner patients. Thoracolumbar fascia was incised in line with skin incisions. In lower lumbar spine (L4-S2), intermuscular plane between multifidus (MF) and longissimus (LM) was identified. In upper lumbar spine (L1-L3), intermuscular plane between pars thoracis (PT) and pars lumborum (PL) was identified (Figure 2). In the lower dorsal spine (D7-D12), the trapezius muscle is the most superficial muscle, and its fibers were split horizontally (Figure 3), and then the intermuscular plane between semispinalis (SM), spinalis (SP), and multifidus medially and longissimus laterally was identified. The plane was identified by visualization; finger dissection was carried out, and Gelpi self-retaining retractors were placed. Additionally, Langenbeck’s retractor was used while placing screws for medial retraction when needed. The facet-transverse groove was palpated. Intersection method was followed. Fluoroscopy was used for confirmation of level and after placing screws. Additionally, fluoroscopic AP view was used for thoracic screws. Before 2018, fluoroscopically confirmed freehand PSF was done. After 2018, O-arm-navigated pedicle screws were inserted in our tertiary center (Figures 4A, 4B). In the Stealth 8 navigation system, intraoperative 3D CT is taken and used for pedicle screw insertion. For non-navigated screws (before 2018 at the tertiary center and still at the peripheral center), we used oblique fluoroscopic views to confirm accuracy. Closure of the wound was done without a drain in layers (Figure 5). In cases of spondylodiscitis or tumor bleeds, a drain was inserted. Postoperatively, the patient was mobilized as tolerated at the earliest, and specific treatment protocol was initiated as per the type of biopsy-guided pathodiagnosis. Regular clinical follow-up and radiographs were taken.

**Figure 2:**
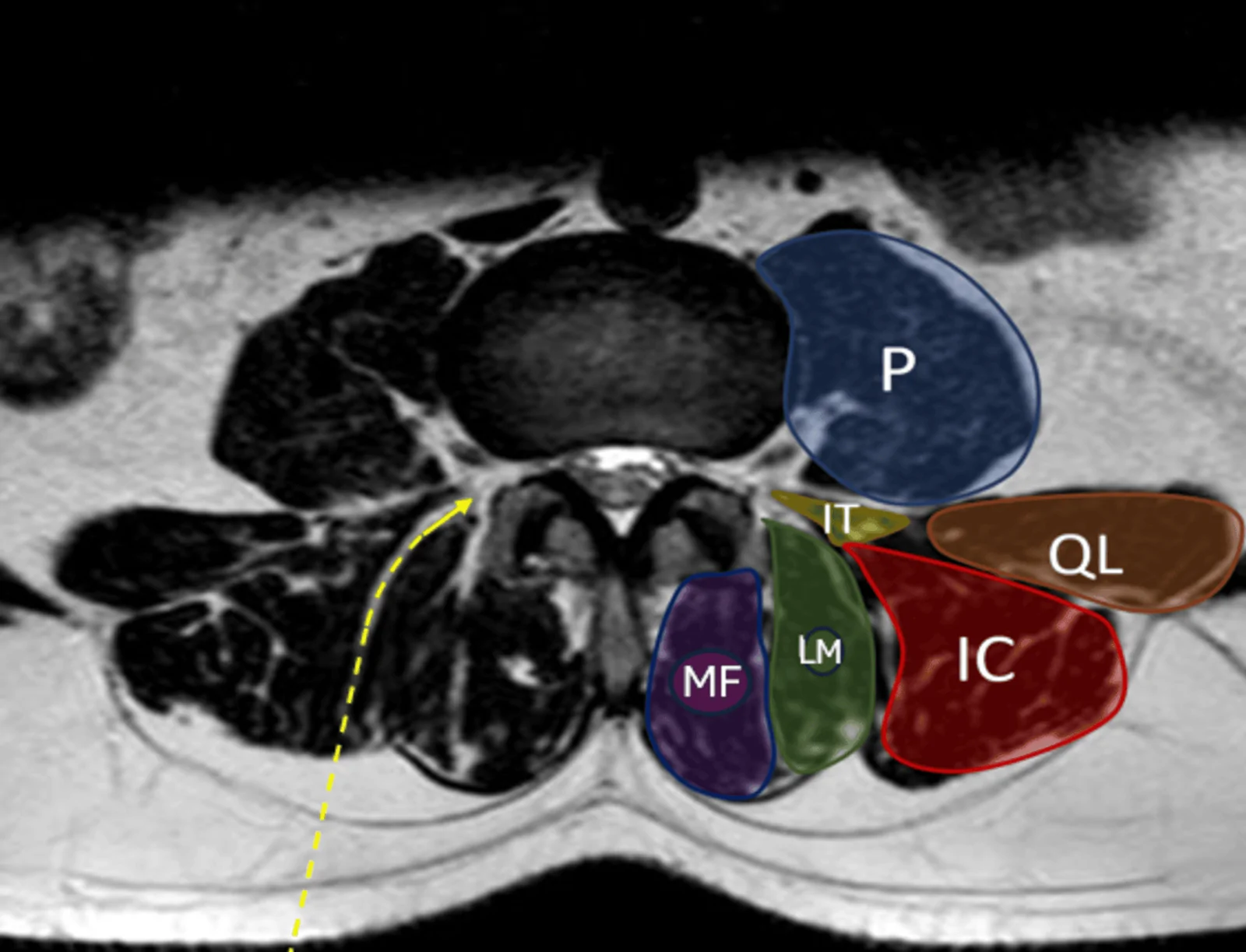
Axial T2-weighted MRI image of the upper lumbar spine. On the right side, the yellow dashed arrow depicts the plane of approach between LM and IC in Weaver’s posterolateral approach. On the left side, the shaded illustration shows multifidus (MF), longissimus (LM), iliocostalis (IC), quadratus lumborum (QL), and psoas major (P). IT: intertransversarii

**Figure 3:**
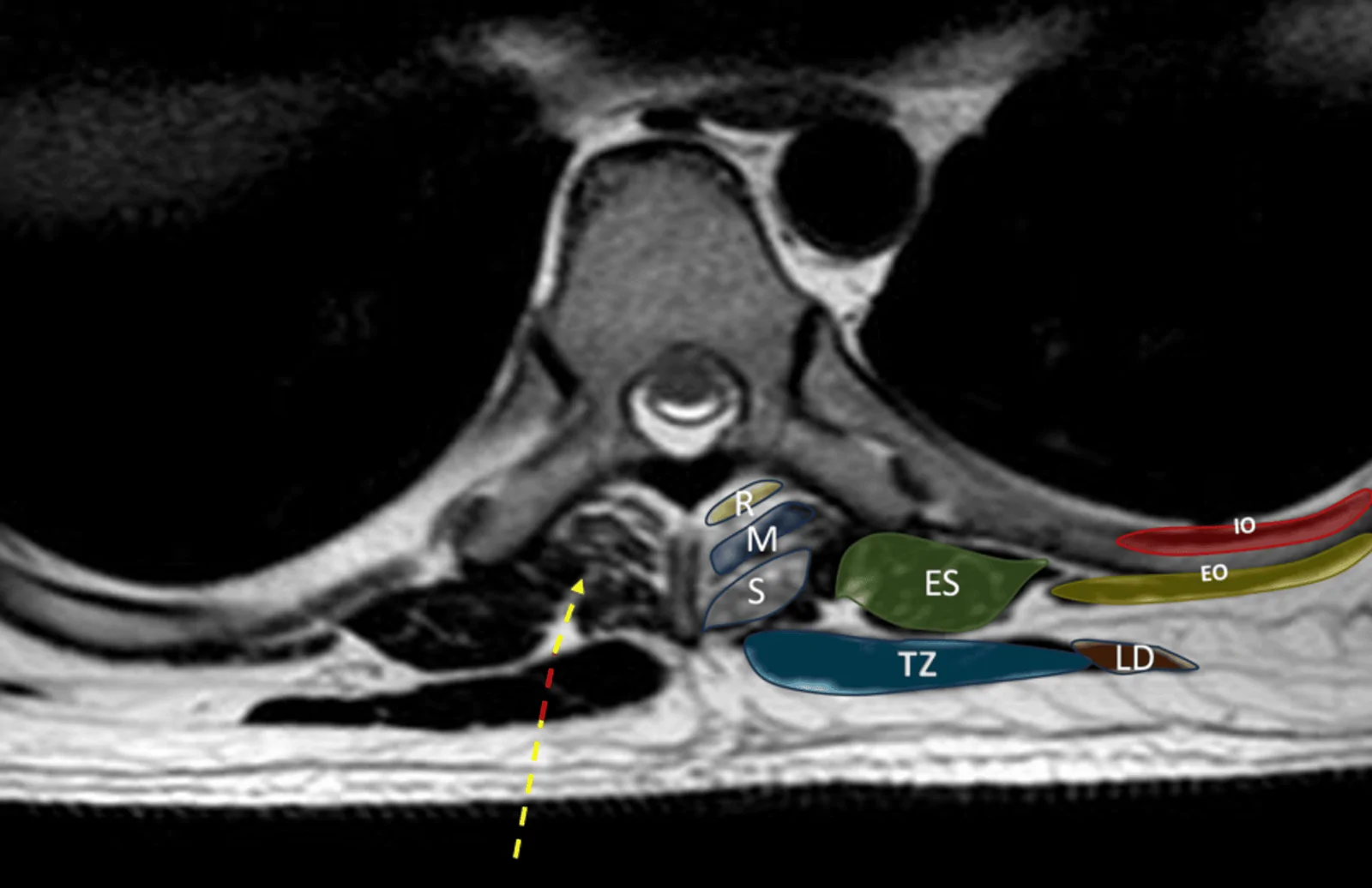
Axial T2-weighted MRI image illustrating shading on the left side involving all muscles of the dorsal spine. Depicted on the right side with the dotted yellow line is the plane of approach in the lower dorsal spine, that is, through the trapezius, splitting its fibers (red broken line), and between the spinalis, semispinalis, and multifidus medially and longissimus laterally. ES: erector spinae; TZ: trapezius; IO: internal oblique; EO: external oblique; R: rotatores; M: multifidus; S: semispinalis; LD: latissimus dorsi

**Figure 4:**
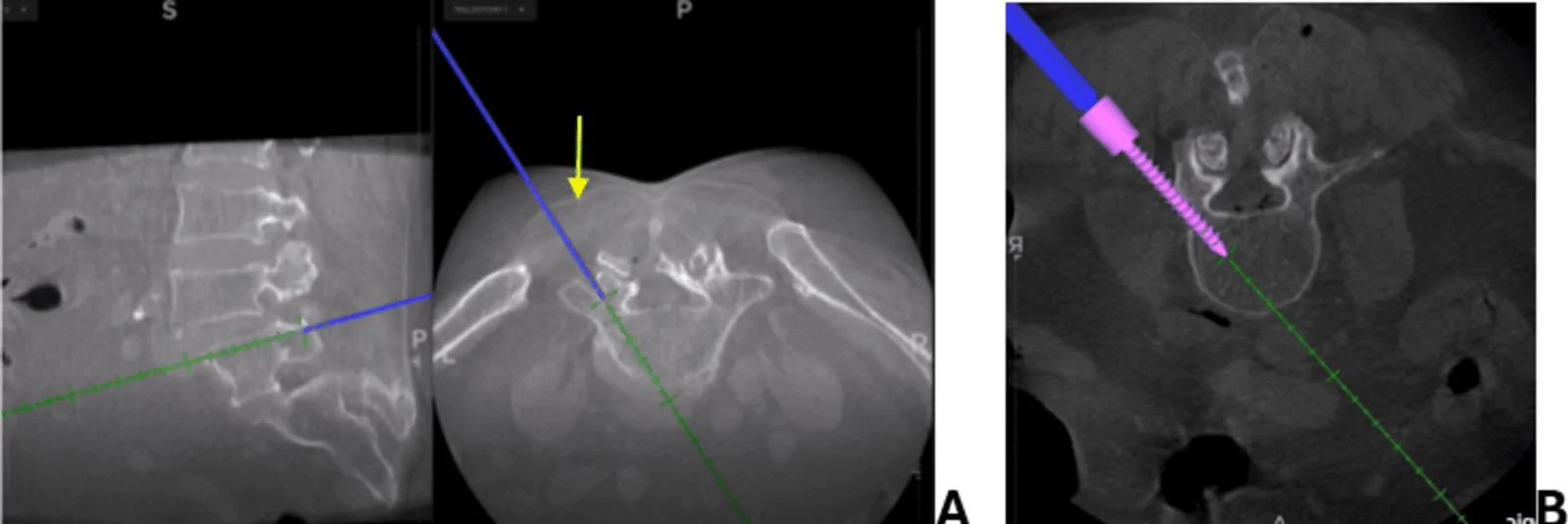
The process of locating the converging O-arm 3D CT-navigated pedicle entry point and pedicle screw insertion. Images A and B show the process of locating the converging O-arm 3D CT-navigated pedicle entry point and pedicle screw insertion, respectively, with the yellow arrow in image A indicating the groove between the multifidus and longissimus. The trajectory is lateral to it, between the longissimus and iliocostalis.

**Figure 5:**
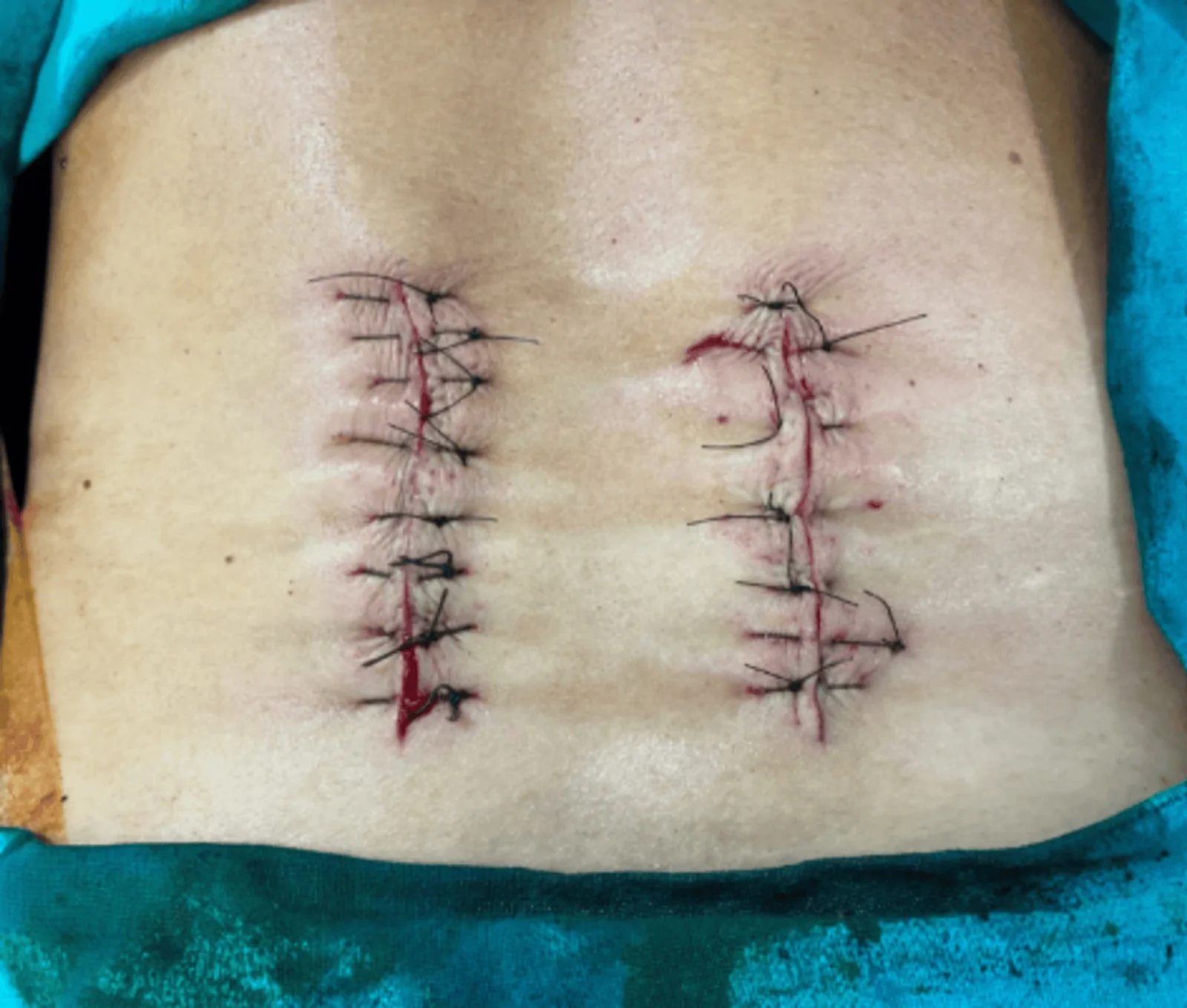
Postoperative wound following layered closure without placement of a drain.

The number of screws placed (navigated or non-navigated) and accuracy of screw placement were analyzed. Grades of breaches, if any, were noted in image intensifier oblique view confirmation or O-arm CT spin if pedicle screws were placed with navigation. Intraoperative estimated blood loss (EBL), operative time of surgery, intraoperative time per screw (total operative time ÷ number of screws), reports of biopsy/tissue culture, length of hospital stay (LOH), requirement of postoperative analgesia, months of follow-up, complications, and any loss of correction/implant status were looked for in final follow-up radiographs. Postoperative analgesics were given as for regular MISS cases for only one day in injectable form, as opposed to COA, for which the institutional protocol is two days. Also, it was noted during the further hospital stay whether the patient needed any extra bolus analgesia beyond the standard protocol of MISS PSF. In this semi-quantitative analysis, we judged it equivalent to COA if the patient needed additional analgesics; if not, it was considered lower and equivalent to MISS, and higher if more than routine analgesics for open surgery were needed. The immediate postoperative and final follow-up radiographs were assessed for segmental Cobb angle, and loss of correction was calculated.

Statistical analysis

Patients' demographics and characteristic categorical variables were analyzed. Mean±SD (minimum and maximum) for applicable variables were calculated. Each category was compared using appropriate statistical tools like Pearson's correlation, unpaired Student's t-test, and paired t-test. Statistical analysis was performed with SPSS version 20.0 (Chicago, IL: SPSS Inc.).

## Results

Demographic results

A total of 47 patients were enrolled in the study with a minimum follow-up of 24 months, except for patients with tumors. The male-to-female ratio was 1.1:1, with 25 males (53%) and 22 female patients (47%). The patients were from all decades of adult life, ranging from 34 to 89 years, with a mean age of 59.1±12.04 years. The sites of involvement were the thoracolumbar vertebrae (n=34, 72.3%) and paradiscal regions (n=13, 27.6%), extending from T9 to L5. Demographic data of patients are tabulated in Table 1. The indications were fracture, tumor, and spondylodiscitis among the spectrum of cases (Figures 6A-6H, 7A-7I, 8A-8H).

**Table 1:** Demographic characteristics of the patients. ASIA: American Spinal Injury Association; TLICS: Thoracolumbar Injury Classification and Severity Score; SINS: Spinal Instability Neoplastic Score

Demographics	Number of patients (n=47)
Male/female	25/22
Age (years)	59.1±12.04 (34-89)
Level	T9 to L5
Indications	
Traumatic fracture (TLICS score 3 {n=0}, 4 {n=3}, 5 {n=15}), n (%)	18 (38.2)
Pathological tumor fracture (SINS score: 13.5+2.6) (11-17), n (%)	6 (12.7)
Fragility fracture (TLICS score: 3 {n=4}, 4 {n=0}, 5 {n=0}), n (%)	4 (8.5)
Infective spondylodiscitis (Pola score: A {n=4}, B {n=6}, C {n=7}), n (%)	17 (36.2)
Andersson lesion (transvertebral=2), n (%)	2 (4.2)
Neurology (ASIA Score)	
D, n (%)	3 (6.3)
E, n (%)	44 (93.6)

**Figure 6:**
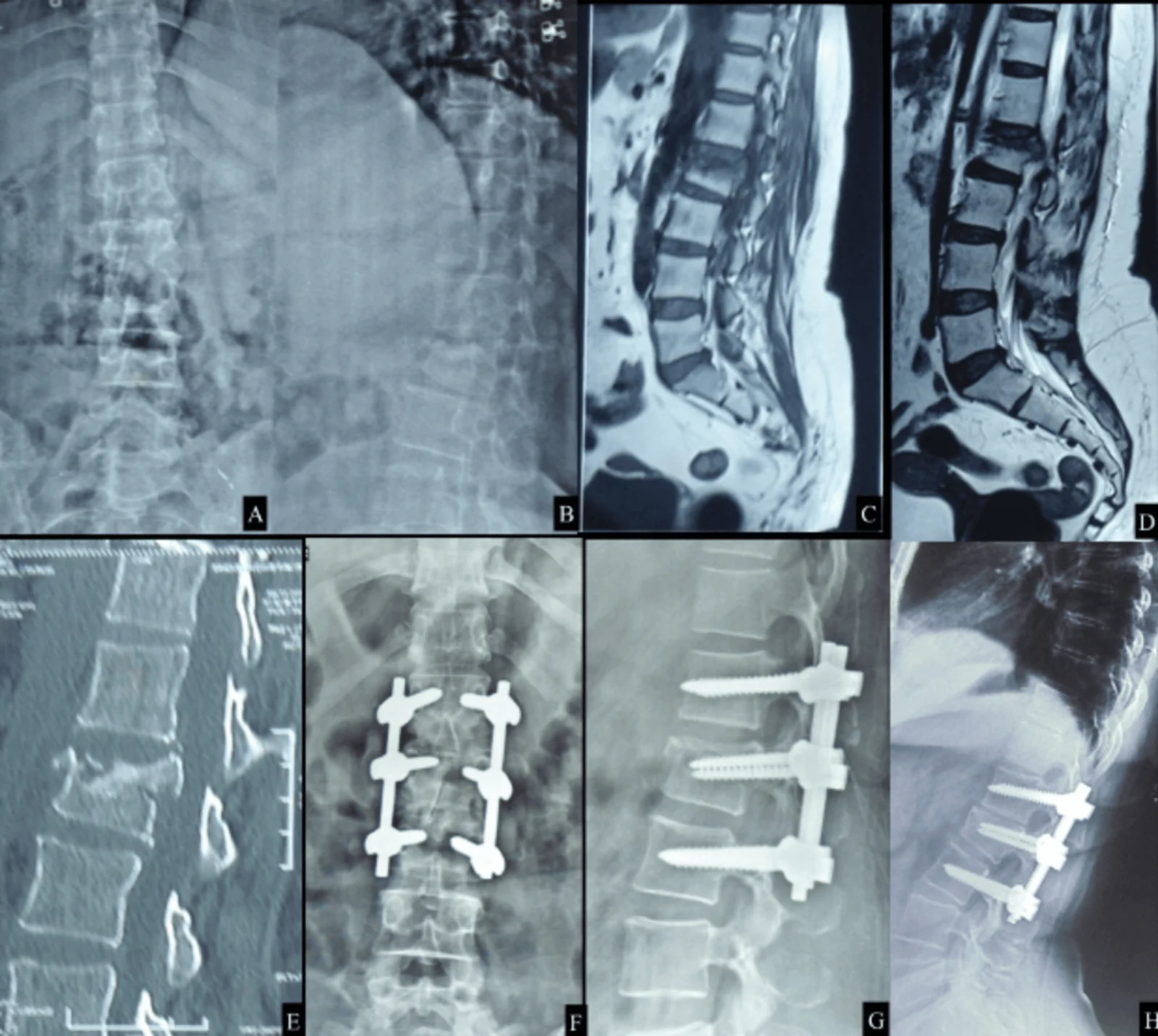
Radiological images of a 37-year-old male with post-traumatic L2 fracture with intact neurology (TLICS score 4) fixed with pedicle screws using posterolateral approach (Weaver Jr’s). Images A and B show preoperative AP and lateral X-rays; C and D show preoperative MRI images; D shows a CT scan; F and G show immediate postoperative radiographs of the patient; and image H shows follow-up healed status with a loss of correction of 3°. TLICS: Thoracolumbar Injury Classification and Severity Score

**Figure 7:**
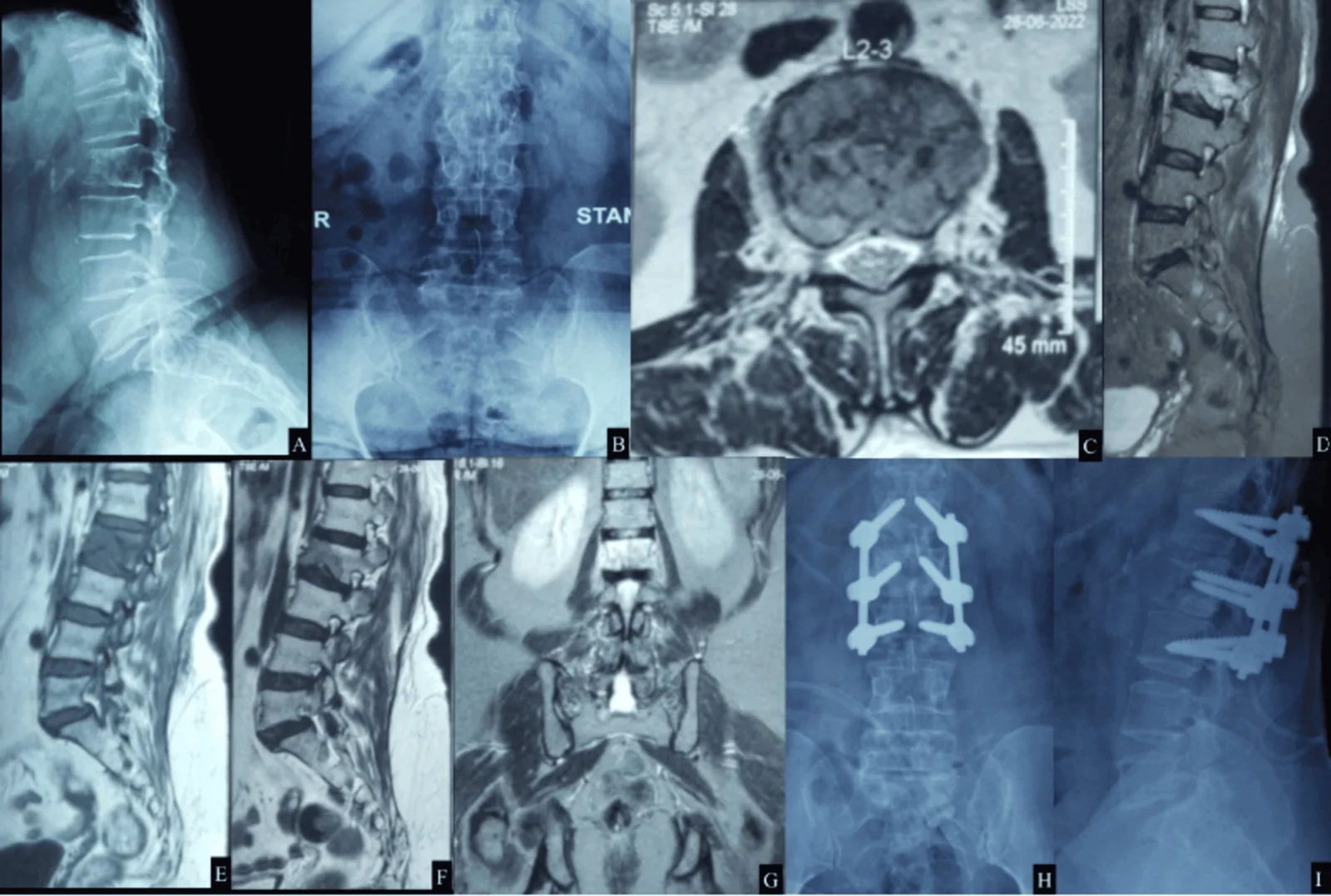
MRI and X-ray images of 48-year-old man with an L2 neoplastic lesion secondary to metastasis and intact neurology (SINS score: 17), treated with short-segment pedicle screw fixation and biopsy. Images A and B show lateral and AP X-ray radiographs, respectively; C-G show axial, sagittal, and coronal MRI images; and H and I showing postoperative AP and lateral radiographic views, respectively, of the patient with L1 to L3 pedicle screw fixation. The patient remained ambulatory before succumbing to metastasis at 25 months. SINS: Spinal Instability Neoplastic Score

**Figure 8:**
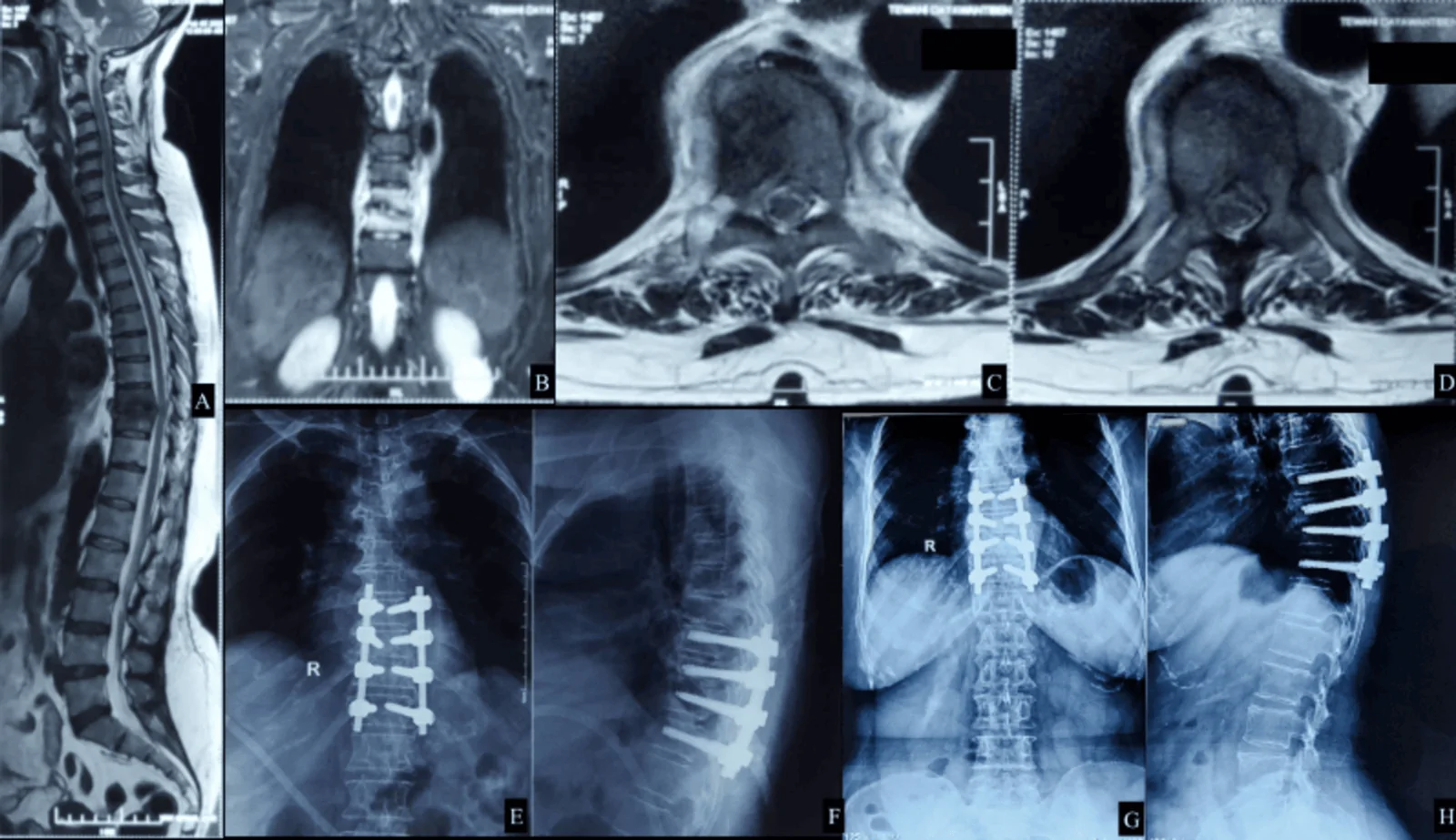
Images of a 61-year-old woman with D9-D10 spondylodiscitis and intact neurology, treated with D8-D11 pedicle screw fixation and biopsy under navigation guidance. Image A shows the sagittal T2-weighted MRI image of the lesion. B shows the STIR coronal MRI image of the lesion. C and D show axial T2-weighted MRI images of the pre- and paravertebral collection. E and F show the postoperative AP and lateral radiographs, respectively, of the patient with D8 to D12 fixation. G and H show healed, maintained follow-up radiographs at 35 months.

Intraoperative variables are tabulated in Table 2. Fluoroscopy-assisted non-navigated pedicle screws were inserted in 24 cases (51%), and O-arm-navigated pedicle screws were inserted in 23 cases (49%). There were no cases of pedicle breach by screws (100% accuracy) noted on fluoroscopic oblique views confirmation or O-arm CT spine.

**Table 2:** Intraoperative variables of the patients.

Intraoperative variables (n=47)	Quantification (n)/average±standard deviation (range)
Total number of screws	289
Number of screws per patient	6.14±1.43 (4-10)
Navigated screws	132, 45.6% (in 23 patients)
Non-navigated screws	157, 54.3% (in 24 patients)
Blood loss (mL) in non-navigated/navigated	44.78±8.45/45.21±8.45 (30-80)
OR time (min) in non-navigated/navigated	61.73±14.9/59.56±11.06 (40-80)
Time per screw (min)	10.11±2.5 (6-17.5)

Intraoperative tissue samples were sent for cultures and/or biopsy in 26 patients (55.3%). Tissue sampling was not required in 21 patients (44.7%) with traumatic thoracolumbar fractures. The pathodiagnostic yield of biopsy samples is summarized in Table 3.

**Table 3:** Pathodiagnostic yield of biopsy samples.

Biopsy and tissue culture results (n=26)	
Type of pathology	Number of cases, n (%)
Tuberculous spondylodiscitis	9 (19.1)
Pyogenic spondylodiscitis	8 (17.02)
Primary tumor (metastatic sarcoma/thyroid carcinoma)	2 (4.2)
Multiple myeloma	2 (4.2)
Osteoporotic osteoid tissue	3 (6.3)
Non-specific inflammation	2 (4.2)

An average loss of correction of 3.46° was noted in 30 patients (63.8%). Most patients (45; 95.7%) had no complications; one case of superficial surgical site dehiscence and one case of sinus tract formation (in a tuberculous patient) were noted. The dehiscence case healed secondarily. The sinus tract healed in due course without any additional intervention. Table 4 summarizes the postoperative outcome variables.

**Table 4:** Postoperative outcome variables.

Parameters	Values
Length of hospital stay (days)	2.46±0.55 (2-4)
Complications	
Nil, n (%)	45 (95.7)
Superficial surgical site infection, n (%)	1 (2.1)
Sinus tract formation, n (%)	1 (2.1)
Loss of correction (°)	3.46±4.92 (0-20)
No change, n (%)	17 (36.1)
Loss of correction, n (%)	30 (63.8)
Follow-up (months)	35.58±12.16 (13-73)

## Discussion

Paraspinal musculature consists of a group of muscles that can be broadly classified as extrinsic and intrinsic muscles. Extrinsic muscle groups are mainly concerned with movement of the appendicular skeleton and are the superficial muscles covering the intrinsic muscles beneath them. Intrinsic muscles lie deep to superficial muscles and are responsible for tone and motion of the spine. Intrinsic muscles can be sub-classified as superficial (longissimus and iliocostalis, which are part of quadratus lumborum), middle layer (multifidus), and deep (intertransversarii and interspinalis group) [12]. Quadratus lumborum (QL) is a broad, quadrangular muscle consisting of various intersecting fibers extending from the anterior surface, lateral surface, and posterolateral aspect of the transverse processes of the lumbar spine and connecting to the ilium, 12th rib, and 12th thoracic vertebra. QL can be broadly classified as iliolumbar fibers connecting transverse processes to the ilium, lumbocostal fibers connecting lumbar transverse processes to the 12th rib, iliocostal fibers connecting the ilium to the 12th rib, and the fourth anterior fascicle connecting the ilium to the body of the 12th thoracic vertebra [12]. Iliocostal (IC) fibers, along with longissimus (LM) fibers, interdigitate to form the erector spinae complex (ESC). IC and LM fibers have two components named pars thoracis (PT) and pars lumborum (PL). PT fibers arise from the pars region of the thoracic vertebrae, span the lumbar vertebrae with attachments medial to the spinous process through the erector spinae aponeurosis (ESA), stabilizing the thorax to the iliosacrum. PL fibers are posterolateral to PT fibers.

PL fibers arise from transverse processes and insert into the ilium [13]. Multifidus is the bulkiest and most medial of all the major paraspinal muscles except for the muscles of the deep layer. Multifidus (MF) is located medial to facet joint and extends up to sacrum. Multifidus takes its origin from laminae, base of spinous processes, and tip of spinous processes. The laminar fibers are the shortest and the thinnest, arising from the caudal end of the dorsal surface of the lamina of each lumbar vertebra and inserting into the mammillary process, just two levels caudal to the vertebra of origin. The major bulk of the multifidus forms from fibers from the spinous process, arranged into five fascicles at each vertebral level. Fibers arise from two attachments, namely from tip of spinous processes and base of spinous processes. The fascicles from tips of spinous processes confluence to form common tendon. Fascicles originating from base of spinous process of L1 insert into mammillary process of L4, and fascicles from common tendon insert into mammillary processes of L5, S1, and posterior superior iliac spine, resulting in interdigitation of fibers [13]. An intermuscular cleavage plane between MF and LM exists throughout the entire length of L1-S1 but varies in its location from the midline. For example, the intermuscular plane in lower lumbar spine (L4-S1) is lateral to upper lumbar spine (L1-L3) owing to bulkier MF in lower lumbar spine. The intermuscular plane between sacrospinalis (LM and IC) and QL, and that between LM and IC, doesn’t exist in the lower lumbar spine (L4-S1) [14].

Various paraspinal approaches have been described in the literature, including Watkins' far lateral approach, Wiltse's transmuscular approach, Weaver Jr.’s lateral intramuscular approach, and a minimally invasive percutaneous approach [7,14-16]. Paraspinal approaches have various advantages. They follow natural anatomical planes, so they require minimal muscle stripping or cutting. The learning curve is shorter, and with minimal training and shorter operative time, paraspinal approaches can provide good visualization of the facet-transverse junction for pedicle screw entry points with a much clearer field. Bleeding is usually not encountered from paraspinal musculature [17]. In 1953, Watkins described a far lateral approach between QL and sacrospinalis (SP), requiring resection of the ilium [15]. In 1960, Wiltse and Spencer described SP splitting transmuscular approach between MF and LM [8].

For more central and contralateral decompression, Wiltse and Spencer described a modification in which LM is retracted laterally, and MF is completely stripped off medial to the facet joint [8]. Initially, Wiltse and Spencer had described these approaches by two separate paraspinal incisions, but due to esthetic concerns, they described several modifications [8]. Wiltse and Spencer, in his modification, described a single midline incision followed by subcutaneous dissection laterally up to intermuscular planes [8]. This modification needed a much smaller midline incision as compared to separate incision length, but led to the risk of creating a subcutaneous potential dead space, which needed to be obliterated by tight closure [8]. In 2007, Weaver Jr described lateral transmuscular approach between PT and PL for lower lumbar spine (L3-S2) [7]. Weaver Jr described that transmuscular plane between PT and PL is more identifiable up to L3, above which PT and PL fibers interdigitate, and their division becomes obscured [7]. This approach was proposed to have the advantage of clearer facet-transverse junction visualization for pedicle screw insertion and the possibility of a more medial trajectory than COA approaches, without much stripping of MF and PT [7]. However, recent studies suggest this transmuscular plane is unavailable between L4 and S2, and that in the lower lumbar spine the LM and IC confluence forms the ESA, which inserts into the posteromedial iliac crest; this plane is available only at the upper lumbar spine (L1-L3) [18].

Our study included all patients who underwent spinal fixations by PLOA in our center. The patients (n=47, 100%) were from all decades of adult life, ranging from 34-89 years, with a mean age of 59.1±12.04 years, and satisfied the inclusion and outcome criteria. Males constituted the majority (n=25, 53.1%) in our sample set. Traumatic cases (n=18, 38.2%) were more common in our sample set, followed by infective cases (n=17, 36.1%). Out of 47, biopsies were sent in 26 cases (55.3%), most of which came out to be tuberculous spondylodiscitis (n=9, 19.1%). The majority of our patients (n=44, 93.6%) had no neurological compromise (American Spinal Injury Association {ASIA} grade E).

The literature has previously reported a number of studies regarding paramedian approaches solely or in comparison to open or percutaneous approaches. The duration of surgery plays an important role in the incidence of perioperative morbidity and complications. The increased probability of facing technical challenges in patients can lead to increased duration of surgery to overcome them [19]. Most studies in the literature reported mean operative times of around 63.3-111.6 min and concluded that the operative time of PLOA is either comparable to or less than percutaneous and COA approaches. Additional time can be attributed to oblique lateral lumbar interbody fusion (OLIF) and lateral interbody fusion procedures. In our study, the mean operative time was 61.73±14.9 min, and 59.56±11.06 min for fluoroscopy- and navigation-guided screws, respectively.

Bleeding and intraoperative blood loss are common issues in spine surgery, but working through intermuscular planes in PLOA may account for a decrease in intraoperative blood loss as compared to the conventional open approach. Lu et al. reported that there was no statistically significant difference in blood loss between PLOA and percutaneous transmuscular groups [3]. In the present study, reported blood loss was 44.78±8.45 mL and 45.21±8.45 mL in fluoroscopy- and navigation-guided screws, respectively.

Accuracy of pedicle screws ranged from 95.2% to 100% for PLOA and 94.5% to 96.8% for the percutaneous group [3]. In our study, we reported no breaches of the inserted pedicle screws, although navigated screws have been reported to increase accuracy in most studies. Fortunately, we also found no gross breaches in non-navigated screws, as assessed on oblique views. This could be attributed to better convergence of pedicle screws due to lateral entry in the facet-transverse groove. This finding of accuracy has also been corroborated in the study by Weaver Jr [7].

COA approach has an advantage over PLOA and the percutaneous approach in exposure of the pedicle screw entry point and better visualization due to complete stripping and a model-like spatial orientation. However, operative time in the COA group is prolonged, which may be due to more soft-tissue dissection and suturing time. The operative time can be further decreased with an increase in the accuracy of PSF insertion in the PLOA group by using navigation and awl-tip tap (Medtronic navigated 4.5-5.5 mm awl-tip tap) for direct screw insertion [20]. As PLOA involves blunt dissection along natural cleavage planes, estimated blood loss (EBL) is lower as compared to COA and similar to or less than that in the percutaneous group. Table 5 summarizes the literature analysis.

**Table 5:** Review of the literature on intraoperative outcome measures. TLF: traumatic thoracolumbar fracture; N/A: not classified; PLOA: posterolateral intermuscular minimally invasive open approach

S. no.	Studies	Etiology	Cases (n)	Approach	Operating time (min)	Fixation levels/patient	Intraoperative blood loss (mL)	Accuracy of pedicle screws (%)	Hospital stay	Complications	Loss of correction at final follow-up
1	Labbe et al. (2018) [21]	TLF	12	Wiltse	111.6±30.3 (65-150)	2:10/3:2	N/A	N/A	3 days shorter than open group	Nil	Nil
			12	Midline intramuscular	97.7±30.1 (55-170)	2:9/3:3	N/A	N/A	N/A	Nil	Nil
2	Jiang et al. (2012) [22]	TLF	30	Wiltse	89.8±16.1 (70-130)	N/A	145±60.7 (50-300)	96.66	10.8±1.4 (9-14)	1 superficial infection. Treated with debridement and antibiotics	2.33±1.69
			31	Percutaneous	79.7±12.7 (60-105)	N/A	79.0±40.4 (501-150)	95.16	9.7±0.9	Nil	3.35±2.70
3	Zou et al. (2020) [19]	TLF	30	Wiltse	65.5±7.5	N/A	53.7±11.0	94.8	4.3±0.7	Nil	N/A
			30	Percutaneous	77.0±7.6	N/A	55.1±13.3	94.5	4.4±0.6	Nil	N/A
4	Pang et al. (2009) [23]	TLF	34	Wiltse	63.3±16.8	N/A	80.1±20.4	N/A	N/A	N/A	Nil
			28	Traditional	65.1±18.6	N/A	280.3±30.8	N/A	N/A	N/A	Nil
5	Lu et al. (2021) [3]	TLF	28	Wiltse	68.1±9.8	N/A	38.8±12.2	95.2	4.2±0.9	Nil	Local Cobb angle (LCA) (1.8±2.9); vertebral wedge angle (VWA) (0.7±1.8)
			26	Percutaneous	76.1±9.0	N/A	36.0±10.7	96.8	4.0±0.9	Nil	LCA: 1.5±2.7; VWA: 0.2±2.7
6	Fan et al. (2017) [24]	TLF	49	Wiltse	63.3±8.9	N/A	55.3±20.8	97.28	4.1±1.0	1 delayed wound healing	N/A
			63	Percutaneous	72.5±7.7	N/A	54.0±17.2	96.56	4.2±0.7	3 delayed wound healing	N/A
7	Our study	Varied pathology	47	PLOA	60.2±13.2	6.14±1.43	45.7±9.9	100	2.46±0.55	Superficial infection (1); sinus tract formation (1)	30 patients (3.46±4.92, 0-20)

Pang et al. reported that the PLOA group had statistically significant advantages in VAS, duration of recumbency, and exercise initiation compared with the COA group [23]. It is hypothesized that patients with PLOA experience less pain and can be mobilized earlier as compared with the COA group because of the intactness of the multifidus muscle [7]. In our study, we divided patients into three categories based on postoperative semi-quantitative analgesic requirement as compared with the COA group. Category 1 consisted of 29 patients (61.7%) whose analgesic requirement was less than that of COA patients and similar to that of MISS fixation surgeries. Category 2 consisted of 14 patients (29.7%) whose analgesic requirement was comparable to that of COA patients. Category 3 consisted of four patients (8.5%) whose analgesic requirement was higher than that of COA patients.

Zou et al. reported that improvement in ODI scores preoperatively and at each follow-up was not significantly different between PLOA and percutaneous groups [19]. This study was not focused on clinical outcome measures, and patients were of different spectra of pathologies; data analysis was not included in the study report. This study was conducted only to re-establish the utility of PLOA for stabilization in different pathologies of spinal instability.

Lu et al. in PLOA and percutaneous groups reported that improvement in correction was statistically significant in both groups, but they were not significantly different between the groups at each follow-up [3]. In our study, we compared immediate postoperative and final follow-up radiographs for Cobb angle and loss of correction. A total of 17 patients (36.17%) had no loss of correction, and 30 patients (63.8%) had loss of correction ranging from 0°-20°, with a mean loss of correction of 3.46°±4.92°.

Nominal healing problems are reported as complications in PLOA by Jiang et al. (n=1) and Fan et al. (n=1) [22,25]. In our study, the majority of patients, i.e., 45 patients (95.7%), had no complications except two, one of which had superficial surgical site dehiscence, and another had sinus tract formation. Both healed in due course without any intervention.

As the PLOA approach involves natural intermuscular planes, major vessels are avoided. Moreover, paraspinal muscles fill the lateral anatomical cavities postsuturing and provide tamponade effect, effectively negating the need for drain insertion [22]. Routinely, postoperative drains are removed on second or third day at our institute, and it is reported in other COA literature series as well [26]. No need for drain insertion and faster mobilization lead to early discharge and early significant improvement in VAS and ODI scores as compared to COA. In our study, the length of hospital stay was 2.46±0.55 days. Introducing PPSF with serial dilators reduces paraspinal muscle injury, but with the existing MISS fixation systems, inserting a rod leads to crushing or entrapment of muscles and soft tissue during insertion and can lead to increased postoperative pain and delayed recovery. Their postoperative course is similar to PLOA group, if not better [20]. Labbe et al. reported that patients in PLOA group had a hospital stay at least three days shorter than open group [21]. Most of the studies report hospital stay duration ranging from 4.0-10.8 days for PLOA. Our length of hospital stay (LOH) was remarkably low at 2.46±0.55 days.

Disadvantages of PLOA are its larger incision and esthetics. For the same reason, among the originators, Wiltse and Spencer themselves had suggested a median superficial approach [8]. Moreover, there is an inability to extend more than four levels usually without changing the natural muscle plane. There is no access to the spine medial to the facet joints for neural decompression in Weaver’s and Watkins’s approach (though possible in Wiltse’s), and also there is an inability to put cross connections. But converging screws themselves increase the pull-out strength [2].

Despite numerous advantages and disadvantages of the posterolateral approaches stated in this study, there are certain limitations to the study. The non-homogeneous group of patients (n=47) included various pathologies and smaller numbers in each category, making the outcomes statistically non-conclusive. The uncontrolled retrospective design can assess feasibility and observed outcomes, but cannot establish comparative superiority or redefine clinical indications. The retrospective nature of the study can give rise to a potential selection bias. The absence of a control group undergoing conventional open approach was not taken. But, it shows the possibility of a less morbid approach to be used when only stabilization is needed, and PPSF cannot be used in cost-sensitive regions of the world. Complications related to the primary disease were not included in the outcome, such as those related to medications for osteoporosis/tumor therapy or antibiotics/tuberculous therapy. The focus in this study was only surgery-related complications. The assessment of breach in fluoroscopy-guided pedicle screws was done by taking oblique views and confirming with another surgeon on the team; no other objective method was used. Moreover, it can be argued that cost-prohibitive 3D CT-based navigation was used when available. However, the authors used it with regular screws in view of the higher screw cost of PPSF. This was possible because, being a private setup, we wrote off gadgetry charges. PLOA is a revisiting of a forgotten art and is better for quicker recovery at the expense of a paradoxically larger incision. But it does follow the true MISS concept of using the natural intermuscular planes.

## Conclusions

PLOA can be considered a safe and effective minimally invasive alternative for thoracolumbar spine stabilization across diverse pathologies, including traumatic fractures, infective spondylodiscitis, pathological tumors, and fragility fractures. The technique offers operative efficiency, reduced blood loss, and acceptable pedicle screw accuracy in both navigated and non-navigated insertions. Early hospital discharge underscores the technique's tissue-sparing advantages over conventional open approaches. PLOA's utility as a cost-effective, lower-morbidity surgical option can be useful, particularly in resource-limited settings where percutaneous technology is unavailable. The preservation of anatomical intermuscular planes validates PLOA as a practical implementation of true MISS principles. However, due to a small sample size and an uncontrolled retrospective study design, these findings are only descriptive and hypothesis-generating. The comparative effectiveness and screw-placement accuracy will require further studies with a controlled study design and standardized outcome assessment.
